# Considerations on the basis of medical reasoning for the use in AI applications

**DOI:** 10.3389/fmed.2024.1451649

**Published:** 2024-09-18

**Authors:** Adamantios Koumpis, Adam S. L. Graefe

**Affiliations:** ^1^Institute for Biomedical Informatics, University Hospital Cologne and Medical Faculty, University of Cologne, Cologne, Germany; ^2^Berlin Institute of Health at Charité – Universitätsmedizin Berlin, Core Unit Digital Medicine and Interoperability, Berlin, Germany

**Keywords:** medical reasoning, artificial intelligence, machine learning, conceptual modeling, abduction, duck test, abstraction, rare diseases

## Abstract

This study discusses the integration of artificial intelligence (AI) and machine learning (ML) in medical reasoning and decision-making, with a focus on the challenges and opportunities associated with the massive consumption of data required for training AI systems, and contrasts this with the limited data typically available to medical practitioners. We advocate for a balanced approach that includes small data and emphasize the importance of maintaining the art of clinical reasoning amid technological advancements. Finally, we highlight the potential of multidisciplinary research in addressing the complexities of medical reasoning and suggest the necessity of careful abstraction and conceptual modeling in AI applications.

## 1 Introduction and context setting: idiosyncrasies of the medical domain

Medicine is, as well-known, *not* an exact science. The widespread adoption of personalized medicine and data-driven approaches provides the opportunity to reconsider the core aspects of medical reasoning and decision-making. Especially with the deployment of artificial intelligence (AI) and machine learning (ML), many paradigms of established medical practices may be challenged and need to be examined in a new light.

For instance, AI and machine learning algorithms need to be “fed” with substantial amounts of data—what is usually called, not always correctly, “big data”— to get them adequately trained. This is *certainly not* the case with humans or even specialists who have limited opportunities to acquire hands-on experience and training by personally accessing larger, let alone vast, amounts of data. Hence, the knowledge acquired during the university years of medical doctors who work in a practice may get evolved with the cases they encounter in the practice, alongside the updates in the medical literature and practice that they can acquire in parallel.

However, it might be interesting to examine the opposite direction of big data by looking into the field of *small data* and how one can ground decision-making practices based on them. The belief that artificial intelligence and machine learning will solve most of our problems is mainly because we think we can afford to optimize the training of as many different models as possible so that the resulting AI systems may function *exactly* as intended by their developers or clinicians, who were involved in the development or the co-development process. However, it is usually the intensity of use and the types and patterns of their usage that may result in suboptimal performance or even malperformance of such a system. Hence, revisiting how medical decisions are made *with small data* makes sense as it helps us better understand the differences between human cognition and machine functioning. Medical doctors are educated not in the way one trains a neural network: it is beneficial to expose medical students to many cases during their studies; however, this exposure by itself does not ensure that they will develop the competence to accurately judge cases later in their career. We only associate richness in experiences and situational training with an increased competence later in their careers.

The positive impact of multidisciplinary research is widely acknowledged, but it is difficult to genuinely achieve in research or professional environments because of the high(er) costs of establishing and maintaining it. Multidisciplinarity is quite wrongly understood to be achieved when people coming from different backgrounds and disciplines are placed together; however, the true value lies in having people who are capable of speaking the same language and, ideally, sharing the same values (1).

In the discipline of law, the term “controlling opinion” is used, which applies to our context (2). This requires courts to *look at all opinions* to determine which is the narrowest compared to others. Controlling opinion can be a mere concurrence rather than a plurality (3). In the case of a medical opinion or decision, one possible way to envision the abovementioned process is by building a “network of nodes” of practices, where individually acquired information can be shared among practices, taking into account data protection, data management, and sharing regulation.

Similar is the case with a tumor board, where teams of experts meet to review and discuss the treatment of cancer patients (4). The goal is to bring together experts from *different* medical specialties to share the unknowns of each different specialization among the teams, such as comparing and bringing together each member's specialized knowledge and applying it to the case of a specific patient. However, the idea in the previous example of controlling opinion is to bring physicians who are routinely making individual decisions regarding the treatment of their patients and to exploit their collective knowledge in terms of building a controlling opinion.

One may see the virtues of this co-creation approach as it is practiced in medical exchange forums such as the American Society of Nephrology—open forum, where members are allowed to share topics for discussion with their peers.

Similarly, the European Clinical Patient Management System (CPMS) is a web-based clinical software application developed for supporting the European Reference Networks (ERNs) on rare, low prevalence, and complex diseases. The system allows the exchange of information between healthcare providers in Europe and is offered for use by healthcare providers who belong to an ERN member hospital. An expert may seek the system for consultation. For this purpose, they need to pseudonymize the name of the patient and substitute it with a nickname, which should have no similarities with the real name of the patient. Other healthcare providers, working in other centers, will only have access to the nickname and their medical data rather than the real data.

Subsequently, the provided data are used for consultation, and thus, the opinions collected reflect the understanding of the experts that is particularly based on the shared information.

## 2 On the essence of medical reasoning

The duck test is a legendary example of abductive reasoning. According to the test, if something *looks* like a duck, *swims* like a duck, and *quacks* like a duck, then it probably is a duck. This reasoning has also been applied in the past in medical cases as discussed in the study conducted by Whiteley (5). Abductive reasoning is not new at all. Rapezzi et al. (6) explicitly mentions that

“the current trend towards mass use of sophisticated diagnostic tools in routine practice — accompanied by a blind faith in technology and predefined diagnostic algorithms — is threatening to kill off the science and art of clinical reasoning.”

He also adds that “[b]esides burning a lot of public and private money to make diagnostic work rather superficial, doctors also risk losing the intellectual pleasure that comes from careful diagnostic reasoning.”

In relation to this, one may relate the aforementioned duck test as a specific instance, which, if related to the increased expectations we now have from AI applications, offers a good example of the manifestation of the problem. However, we see that instead of regarding this as a technology side issue (i.e., considering the *solutions space* that AI technology can offer to users), one needs to see this as a design problem that should be related to the *problem space*, in terms of regarding the AI technology as a means to better understand the problems. This perspective aligns with what Rapezzi et al. (6) referred to as the “science and art of clinical reasoning” which, in the scope of this article, is referred to as medical reasoning.

Especially the fact that these concerns appeared almost 20 years before, when the proliferation or even the emergence of artificial intelligence tools and platforms was anything but obvious or granted, makes the case even more relevant. In the bibliography, other relevant research can also be found. For instance, the study by Rejón Altable (7) examines what the author terms “clinical judgment” (in our paper referred to as medical reasoning) as an abductive inference. The author situated the research in the field of psychiatric semiology, where he built the case for the need to foster a “careful balance between the information present in descriptive definitions and the information absent from the definition but present in singular symptoms.” In our article, this is the role that we expect from the deployment of the duck test. According to Rejón Altable (7), “general abductive inference and common clinical practice are retroductive”—essentially what one would consider an *educated* guess. It is at this point exactly that one considers that medical education, which takes several years for humans to acquire, cannot be replaced by technologies that are only meant to *complement* human skills and assist them in performing their tasks.

In addition, Rejón Altable's “not-in-definition” material may be relevant enough to be taken into account especially when considering the missing information that an AI system cannot capture. Wilson appealingly mentions that “one might argue that, even if not all abduction is generative, and even if not all abduction is inferential, all abduction still results in *hypotheses*” (8). However, in times when we experience an increasing attraction to generative AI, with the growing interest in topics related to, among others, end user-driven application of generative AI models in healthcare and the use of multimodal data to advance generative AI applications in the field of biomedical research, it is obvious that aspects of relevance and causal relationships among the variables of interest will continue to be important aspects of medical reasoning that are hard to implement in automated systems. This is nothing new, and one can even revisit the legendary and pathbreaking work of Feinstein (9). In his work, Feinstein identifies the (growing) gap between “bedside” medical practice and the increasing role of laboratory research. At present, one may understand or interpret that Feinstein was referring to medical reasoning as a “bedside” experience and the one that is guided by the results of some AI-supported procedure as the increasing role of laboratory results.

The Liskov Substitution Principle (10) in computer science is sometimes expressed as a counter-example to the duck test. The principle suggests that if something *looks like a duck* and *quacks like a duck* but needs batteries, we may probably have the *wrong abstraction*.

The latter may appear like “sending the ball out of play,” but it may be worth having a closer look. Conceptual models, as we know well, are abstractions of things in the real world, whether physical or social. This holds true not only in medicine but also in other fields, such as economics, sociology, and even forecasting. The success or failure of these models depends partly on how well they represent the real world. Their computability and a number of other parameters are also relevant and important.

Below, we illustrate the case with two example cases, which we used as the source bibliography.

Example case 1: In Case 1, a 16-year-old male patient with no known previous illnesses, except for pneumonia 3 years ago, was hospitalized after severe acute diarrhea and dehydration. He was diagnosed with infectious bacterial diarrhea caused by *Campylobacter enterocolitis* and showed evidence of splenomegaly and lymphoproliferation. Further testing excluded any malicious diseases that could have caused this.

After further anamnesis, recurrent respiratory infections since a young age, often leading to otitis media and sinusitis, were documented.

A CT scan also revealed evidence of bronchiectasis, which led to a diagnosis of activated phosphoinositide 3-kinase (PI3K) delta syndrome (APDS), as reported by Ewertowska et al. (11). Based on this article, we modeled the example clinical case.

Example case 2: In Case 2, a 26-year-old female patient with previous diagnoses include neonatal cholestasis, chronic diarrhea from infancy, a cataract diagnosed at the age of 8 years, and depression for 1.5 years diagnosed at the age of 22. Given her current symptoms, she was diagnosed with peripheral neuropathy.

By revisiting the methodology, particularly through the duck test, and questioning the appropriateness of the abstraction followed to date, we may identify cerebrotendinous xanthomatosis (CTX) as a potential diagnosis. CTX is a rare disease that affects patients' ability to metabolize fats, specifically cholesterols, based on the model presented in the study of Saussy et al. (12).

According to Orphanet, which is the database of rare diseases, CTX is a condition for which “more than 300 patients have been reported worldwide” (13). The single data points, i.e., symptoms or clinical presentations, individually, are sometimes very common and sometimes highly suspicious. For example, neonatal cholestasis may be indicative of many diseases and often has no pathological value. However, a cataract at the age of 8 is already more suspicious. These combined data points may result in a high sensitivity for these diseases.

Thus, if not looking for the “batteries,” as mentioned above for the adapted version of the Liskov Substitution Principle, which in these cases are the underlying mutations causing these diseases, one *may not find the right abstraction to look for*. This is also compared to Poppers' (31) black swan theory: only because you cannot see a black swan, it is not a proof of its non-existence.

The challenge for medical professionals lies in combining these data points easily. Currently, most data lie in doctors' letters without adhering to fair principles, which make them inaccessible. Thus, especially in the case of rare diseases, machine learning or AI may be *destined to fail* when it comes to accessing reliable data to support clinical decision-making. Furthermore, even if a diagnosis can be made, the lack of diagnosed cases and thus imprecise phenotypic predictions based on a genetic disease further complicate this issue. Clinical predictions that a doctor can give to a patient are mostly imprecise as genetic diagnoses lack precise phenotyping due to missing precise data for deep phenotyping (14).

It is worth mentioning that data points can also be, apart from a symptom, a digital image that has been used to train an algorithm, especially because it has contributed to the formation of a medical decision. In addition, as mentioned before in Section 1, it is important to understand the differences between human cognition and machine functioning and how human cognition works with massive amounts of data (points). An ingenious application of a similar approach can be found in a previous study (15), which implemented the concept of prototypical patients. This approach facilitates learning from the prototypical characteristics of diagnoses in previous cases.

## 3 Conclusion and a note on differential diagnosis

The integration of AI and ML into medical practice has become a rapidly growing field with significant implications for the future of healthcare delivery. When one considers the “*Treachery of Images*” (30), one of the most well-known and, undoubtedly, emblematic paintings of the painter René Magritte, one may observe parallels with the challenges posed by the proliferation of artificial intelligence in our lives. Similar to Magritte's painting, the results of an AI system that has been trained to “parse” images of human lungs to detect cancers, and the training that such an AI system has received to become capable of identifying a tumor and profess at will or discretion on its nature, i.e., whether it is benign or malignant, reflect processes applied to the images, not the disease itself. However, we tend to relate images with diseases. In this context, an entire team of medical experts and specialists will look at an image of a human lung and, depending on their respective expertise and context, build an opinion.

A problem related to AI systems is the massive consumption of information, e.g., the forms of images that are neither subject to relevance nor provenance aspects. In a recently published article, the authors referred to what they call the “last piece of a background puzzle” regarding human oversight of AI systems. They mentioned, equally, elegantly, and correctly, that “for now, at least, AI systems are typically kept on quite a short leash, frequently limited to giving advice to health-care providers who are very much still the final decision-makers” (16). During the review process, we were notified that the discussion should be on the difference between learning processes, on the one hand, and cognitive abilities, on the other hand. There is a considerable scope for interactions between them, but one should be able to see the difference.

Below, we share some thoughts that, individually seen, may not be innovative or new, but when seen together, these thoughts may provide some insights for preparing better for the future of AI-assisted medical reasoning.

For people studying medicine, there is a corpus of bibliography dedicated to “medical thinking,” which aims to introduce future medical doctors to the reasoning processes of their profession, as mentioned by Patel et al. (17), Elstein et al. (18), Graves et al. (19), and Fuks et al. (20). One may always care to consider if one treats the disease or the person (who happens to have a disease or a condition) in medicine. In addition, one may wonder whether one matches patients to diseases, or the other way round, i.e., diseases to patients. In a similar manner, one may wonder whether theories are case-driven and validated or rather the opposite, where they are built based on the cases studied and validated. In many cases, the term “reverse engineering” could be used, and this term is also relevant when considering ongoing discussions regarding both explainability and reproducibility of AI programs, as discussed in the works of Gunderson and Kjensmo (21) and Gunderson (22, 23).

Differential diagnosis is defined in the works of Lamba et al. (24) as “the process of differentiating between probability of one disease vs. that of other diseases with similar symptoms that could possibly account for illness in a patient.” The term and the history of applying the procedure dates back to more than 100 years now. One may see that since the term was introduced by French (29), its relevance has persisted even today with the proliferation of data- and AI-driven medicine.

A process that was conceived to be used by humans may—due to its algorithmic nature—seem to completely suit the needs of machines. This observation highlights the need to educate people about exploring different options in parallel or concurrently. For example, one condition may be eliminated as there is evidence that speaks against it or there is a lack of evidence. Similar to a court procedure, *not guilty* is not the same as *innocent* and the effort of the court is to prove if a person charged for a crime should be declared *guilty* or *not guilty*. One may also draw an analogy to running several court actions in parallel or concurrently for the application of differential diagnosis, aiming to determine which diagnoses are viable.

A concern raised regarding the use of machines in healthcare, as anecdotally noted by Pearn and attributed to Professor Cox, is the idea that “when a doctor gets his teeth into a diagnosis, he may be reluctant to let it go, even when incorrect” (25).

Patel et al. (17) stated that (emphasis is ours) “in the clinical sciences, *the patient is seen as an exemplar to which generalizations based on multiple overlapping models are applied* from disease mechanisms (e.g., physiological, biochemical, pathological) and from the population of similar patients (e.g., typical diagnostic categories described in clinical medicine).” This is where we actually started from, from the duck test as a tool to validate not only the appropriateness of the provided answer but also as a means and an instrument to check the appropriateness (to avoid using the term correctness) of the abstraction followed.

With the proliferation of medical AI systems, one may need to be able to go beyond the technicalities of the particular systems and try answering questions, such as which role should AI systems play in medical reasoning? In addition, cases where AI-based reasoning may come to its limits and offer examples of pitfalls of using AI systems in medical reasoning include, among others, areas such as gender bias (26), the implementation of geriatric medicine (27), the diagnosis and treatment of psychosomatic syndromes (28) and, finally, considerations of cultural, multicultural, or cross-cultural medicine (32).

## Data Availability

The original contributions presented in the study are included in the article/supplementary material, further inquiries can be directed to the corresponding author.
